# The implementation of integrated disease surveillance and response in Liberia after Ebola virus disease outbreak 2015-2017

**DOI:** 10.11604/pamj.supp.2019.33.2.16820

**Published:** 2019-05-28

**Authors:** Thomas Nagbe, Jeremias Domingos Naiene, Julius Monday Rude, Nuha Mahmoud, Mohammed Kromah, Jeremy Sesay, Okeibunor Joseph Chukwudi, Mary Stephen, Ambrose Talisuna, Ali Ahmed Yahaya, Soatiana Rajatonirina, Musoka Fallah, Tolbert Nyenswah, Bernice Dahn, Alex Gasasira, Ibrahima Socé Fall

**Affiliations:** 1 National Public Health Institute, Monrovia, Liberia; 2 World Health Organization, Monrovia, Liberia; 3 World Health Organization, Regional Office for Africa, Brazzaville, Congo; 4 Ministry of Health, Monrovia, Liberia

**Keywords:** Integrated diseases surveillance and response (IDSR), Liberia, Surveillance system, International health regulation (IHR)

## Abstract

**Introduction:**

Although Liberia adapted the integrated diseases surveillance and response (IDSR) in 2004 as a platform for implementation of International Health Regulation (IHR (2005)), IDSR was not actively implemented until 2015. Some innovations and best practices were observed during the implementation of IDSR in Liberia after Ebola virus disease outbreak. This paper describes the different approaches used for implementation of IDSR in Liberia from 2015 to 2017.

**Methods:**

We conducted a cross-sectional study using the findings from IDSR supervisions conducted from September to November 2017 and perused the outbreaks linelists submitted by the counties to the national level from January to December 2017 and key documents available at the national level.

**Results:**

In 2017, the country piloted the use of mobile phones application to store and send data from the health facilities to the national level. In addition, an electronic platform for acute flaccid paralysis (AFP) surveillance called Auto-Visual AFP Detection and Reporting (AVADAR) was piloted in Montserrado County during the first semester of 2017. The timeliness and completeness of reports submitted from the counties to national level were above the target of 80% stable despite the challenges like insufficient resources, including skilled staff.

**Conclusion:**

IDSR is being actively implemented in Liberia since 2015. Although the country is facing the same challenges as other countries during the early stages of implementation of IDSR, the several innovations were implemented in a short time. The surveillance system reveled to be resilient, despite the challenges.

## Introduction

Public health surveillance systems are crucial for detection of unusual trends of diseases or public health events. However, it requires skilled staff, good information system and good laboratory capacity for cases confirmation [1]. Weak surveillance systems, unable to detect early public health emergencies including early stage of outbreaks are public health challenges in several african countries. This weakness contributed to a late recognition of Ebola virus disease (EVD) outbreak in West Africa in 2014, where Liberia, Sierra Leone and Guinea were the most affected countries [2-5]. The World Health Organization (WHO) framework for monitoring and evaluating surveillance and response systems for communicable disease with core and support functions, from which surveillance indicators were derived was adapted by several countries including Liberia [6]. Liberia adapted the integrated diseases surveillance and response (IDSR) in 2004 as a platform for implementation of International Health Regulation (IHR (2005)). Although some progress has been observed over the years, the poor health care facility (HCF) reporting and the inadequate response remained challenges that became obvious after the EVD outbreak [7]. Nevertheless, the outbreak was a good opportunity to strength the surveillance systems in Liberia, and staff were trained, new laboratories were opened and EVD surveillance was also strengthened at the community level [8, 9]. After the control of EVD, the implementation of IDSR was focused not only on case detection but also on response to other priority diseases and public health events [7]. The strengthening of IDSR is part of the investment plan to build a resilient health system in Liberia [10]. Some innovations and best practices were observed during the implementation of IDSR in Liberia after EVD outbreak, as well as dramatic improvement on case detection and response to several outbreaks and public health events. This paper describes the different approaches used for implementation of IDSR in Liberia after EVD outbreak, innovations, best practices and lessons learned.

## Methods

### Setting

Liberia is one of west african countries with a population of 3,489,072 and a population density of 93 people per square mile according to the census 2008 [11]. The country is divided into 15 counties and five main regions, namely [12]: 1) North Western: Bomi, Gbarpolu and Grand Cape Mount counties; 2) South Central: Grand Bassa, Margibi and Montserrado counties; 3) North Central: Bong, Lofa and Nimba counties; 4) South Eastern A: Grand Gedeh, River Cess and Sinoe counties; 5) South Eastern B: Grand Kru, Maryland and River Gee. Montserrado County alone has 33% of population of the entire country and is classified as a very densely populated county, while the south eastern and Gbarpolu counties have small and sparse population [11]. The 15 counties are divided into 90 districts in the country. Due to the huge population, Montserrado County is further divided into 22 health zones.

### Study design

We conducted a cross-sectional study in December 2017 using the IDSR supervisions secondary data collected from September to November, 2017. A supervision checklist was used by the district surveillance officers (DSOs) and zonal surveillance officers (ZSOs) to supervise monthly all the 773 HCFs across the country as part of their routine activities. The supervision checklist was completed using the information provided by the head of the health facility. Besides the review of patient charts, ledgers and lab records, the DSOs and ZSOs also observed directly the presence of guidelines, case definitions and recording and submission of reports. However, from September to November, 2017, 384 HCFs (50%) were supervised at least once and the data used in our analysis (Table 1). The supervision checklist included 65 variables and the qualitative question “What are the major areas for improvement in surveillance activities in this HCF?”. The outbreaks line lists submitted by the counties to the national level from January to December 2017 were used to analyze the outbreaks investigated and the respective IDSR performance indicators. We organized and analyzed the findings according to IDSR core functions, namely case detection, case registration, case notification, data management, data analysis, outbreak preparedness, outbreak detection, outbreak response and feedback, as well as support functions, namely supervision, training, laboratory function, resources and coordination suggested by WHO [6, 13]. We also perused key documents available at the national level, namely, two IDSR technical guidelines for Liberia, IDSR bulletins published at the National Public Health Institute of Liberia (NPHIL) website, outbreaks investigations reports and the Lab records available at NPHIL database from January to December 2017.

**Table 1:** Performance of integrated diseases surveillance and response selected indicators, Liberia September to November, 2017

Integrated disease surveillance and response function	**Liberia Target (%)**	**%Southeastern A (n=29)**	**%Southeastern B (n=16)**	**%North Central (n=113)**	**%South Central (n=162)**	**%South Western (n=64)**	**%Liberia (n=384)**
**Case detection**							
Community simplified case definitions pinned on wall	80	88	92	97	89	92	92
IDSR Standard case definitions pinned on wall	80	93	97	98	93	92	95
Alert and epidemic threshold charts updated within the past 1 month	80	70	70	67	62	83	70
**Case registration**							
Laboratory registers	80	98	87	79	68	80	82
HMIS monthly reporting forms with a correctly filled IPD/OPD section	80	100	100	96	98	98	99
Facility patient registers	80	100	100	100	100	96	99
IDSR case alert and lab submission forms	80	98	95	99	95	95	96
Community trigger and referral forms	80	70	100	77	81	88	83
Case review forms for maternal and neonatal death	80	65	86	72	74	57	71
**Outbreak preparedness**							
IPC protocols available	80	93	64	85	76	91	82
**Outbreak detection**							
Line list of alerts and rumors from the community	80	60	50	36	36	50	46
**Trainings**							
Surveillance Focal Person trained in the IDSR in the past 1 year	80	27	78	29	42	56	46
HCFs with staff trained to collect oral swab	80	95	67	89	36	93	76
CHVs/CHAs trained in CEBS in the catchment area in the past 1 year	80	100	97	84	47	90	84
**Supervisions**							
% HCFs supervised^a^	80	37	26	60	44	89	50
HCFs that received Supervision feedback report from district/county in the last four weeks	80	95	97	92	87	95	93
HCFs conducting supervisory visits to the CHVs and CHAs	80	97	86	77	60	90	82
**Resources**							
Means of communication to communities to the district	80	89	100	99	86	97	94
Adequate and functioning transport, including fuel, for surveillance activities	80	53	82	53	20	80	58
**Standards and guidelines**							
IDSR Technical Guidelines available	80	67	50	57	60	96	66
SOPs for dead body management available	80	66	86	74	57	87	74
The lab SOPs for specimen collection, packaging, and storage for priority diseases available	80	57	92	67	79	85	76

### Data analysis

**Qualitative data:** all the qualitative data from supervision checklist (opinion of the health workers about the major areas for improvement in their HCF) were stored in Microsoft™ Excel. We organized all the answers manually to fit into each of the IDSR functions. The qualitative data obtain through direct observation and relevant reports were recorded as a text and organized according to the IDSR functions.

**Quantitative data:** the quantitative data from supervision checklist were also stored and analyzed in Microsoft™ Excel. From the 65 indicators, we included in analysis only the data fitting in the IDSR functions and with information available from all the 384 HCFs assessed.

**Ethical considerations:** the IDSR activities were part of the routine surveillance and response activities in Liberia. Therefore, no ethical approval was required. The secondary data analysis and publication was authorized by the National Public Health Institution of Liberia. No personal information and confidential data was disclosed.

## Results

**Structure of integrated diseases surveillance and response in Liberia:** poliomyelitis, meningitis, cholera, shigellosis, Lassa fever, measles, rabies, viral hemorrhagic fever, neonatal tetanus, yellow fever, maternal deaths, neonatal deaths and unexplained cluster of deaths or health events were the priority diseases and events to be reported weekly in Liberia under IDSR. In Liberia, the IDSR data were collected first from the communities and points of entry and submitted subsequently to the HCFs, district, county and national levels. In 2017, 88% (679/773) of the facilities were classified as clinic, 7% (55/773) health centers, 5% (38/773) hospitals and < 1% (3/773) health posts. The majority of the HCFs [40% (290/773)] were concentrated in Montserrado County, 83% (240/290) of them classified as private. The surveillance system in Liberia received regular reports from 96% (285/296) of the private HCFs across the country. At the community level, the community health assistants and community health volunteers were responsible for surveillance system and were supervised by community health services supervisors (CHSSs) from the HCFs.

### Core functions

**Figure 1:**
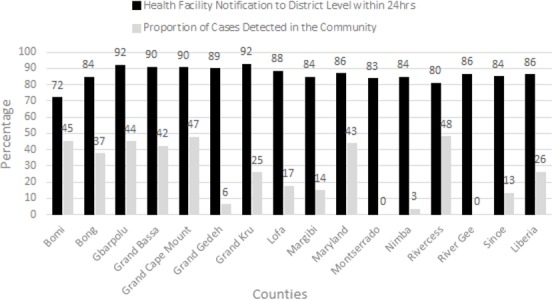
Integrated diseases surveillance and response performance indicators for outbreak detection and notification from the health facilities and communities, Liberia, 2017

**Case detection:** the case definitions of the priority diseases were simplified into a non-technical language and distributed to the community volunteers. During the 2017 assessment, the community case definitions were also pined on the walls in 92% (353/384) of HCFs (Table 1). In addition, 95% (349/384) of the HCFs had pinned on wall standard case definitions and the alert and epidemic threshold charts were present in 70% (369/384) of HCFs. The number of cases of priority diseases detected in the community represented 26% (100/384) of all the cases (Figure 1). Some officers in charge (OICs) disclosed that they do not use the case definitions due to limitations in understanding them and understand the thresholds. Other OICs highlighted the early detection of priority disease will improve with more awareness about the priority diseases at the community level and with active case search at community and facility level.

**Case registration:** the cases were recorded first in the patient’s charts with clinical data and later transferred to the patients ledgers which included all the diagnosis in the HCFs. There were both adults and under-five years old patients ledgers. A recorder was responsible for filling the IDSR ledgers daily and make sure no priority disease were missed. During the 2017 assessment, all the indicators performed above the target of 80% except the case review forms for maternal and neonatal death that were present in 71% (269/384) of the HCFs (Table 1). The need of more reporting tools was also pointed by an OIC, as a challenge, while the other OIC disclosed that usually forgets to send the zero reports.

**Case confirmation:** the peripheral clinics were equipped to perform rapid diagnostic test for malaria, typhoid fever, human immunodeficiency virus, syphilis and hepatitis B and urinalysis. Some clinics could also do microscopy for tuberculosis, malaria and stool tests. The referral hospital could perform gram test for meningitis, microscopy for malaria and stool test. The regional Labs could test for viral hemorrhagic fever like EVD, yellow fever and lassa fever. From January to December 2017, the country was able to confirm outbreaks of seven different conditions (Table 2).

**Table 2:** Outbreaks investigated with cases confirmed by laboratory, Liberia, January to December, 2017

	**South Eastern A**			**South Eastern B**			**North Western**			**South Central**			**North Central**			**Liberia**		
	**Cases**	**Deaths**	**CFR (%)**	**Cases**	**Deaths**	**CFR (%)**	**Cases**	**Deaths**	**CFR (%)**	**Cases**	**Deaths**	**CFR (%)**	**Cases**	**Deaths**	**CFR (%)**	**Cases**	**Deaths**	**CFR (%)**
**Shigellosis^a^**																		
Total Cases	74	0	0.0	11	0	0.0	14	0	0	107	0	0.0	18	0	0.0	224	0	0.0
Lab Confirmed	5	0	0.0	0	0	NA	1	0	0	6	0	0.0	2	0	0.0	14	0	0.0
**Human Rabies^b^**																		
Total Cases	146	2	1.4	80	1	1.3	70	0	0	709	0	0.0	284	0	0.0	1289	3	0.2
Lab Confirmed	0	0	NA	0	0	NA	0	0	NA	1	1	NA	0	0	NA	1	1	100.0
**Cholera^a^**																		
Total Cases	28	0	0.0	16	0	0.0	6	0	0	28	3	10.7	12	2	16.7	90	5	5.6
Lab Confirmed	1	0	0.0	1	0	0.0	0	0	NA	0	0	NA	0	0	NA	2	0	0.0
**Lassa fever**																		
Total Cases	2	0	0.0	0	0	NA	0	0	NA	18	6	33.3	41	13	31.7	61	19	31.1
Lab Confirmed	0	0	NA	1	0	0.0	0	0	NA	11	1	9.1	17	10	58.8	29	11	37.9
**Measles**																		
Total Cases	35	0	0.0	18	0	0.0	77	0	0	317	1	0.3	305	0	0.0	752	1	0.1
Lab Confirmed	5	0	0.0	3	0	0.0	5	0	0	96	0	0.0	110	0	0.0	219	0	0.0
**Meningococcal Disease**																		
Total Cases	1	1	100.0	15	1	6.7	3	0	0	7	3	42.9	6	0	0.0	32	5	15.6
Lab Confirmed	13	9	69.2	0	0	NA	0	0	NA	2	1	50.0	0	0	NA	15	10	66.7
**Rubella^c^**																		
Total Cases	33	0	0.0	11	0	0.0	47	0	0	120	0	0.0	104	0	0.0	315	0	0.0
Lab Confirmed	33	0	0.0	11	0	0.0	47	0	0	120	0	0.0	104	0	0.0	315	0	0.0

a The outbreak was not declared because the epidemic threshold according to the national IDSR guidelines was not reached;

b Besides one lab confirmed case in France, the cases that ended in death were confirmed clinically;

c The outbreak was identified through labs tests of the suspected measles cases. Sinoe and Bomi counties did report any measles conformed cases but detected rubella confirmed cases.

**Figure 2:**
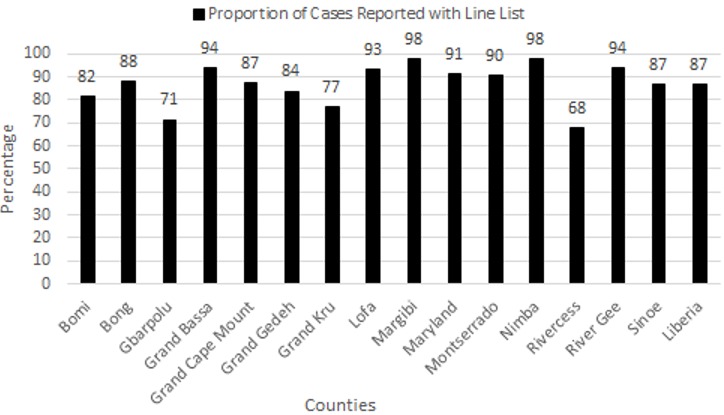
Integrated diseases surveillance and response performance of proportion of cases reported with Line list, Liberia, 2017

**Case notification:** the priority diseases were reported on weekly basis to the immediately next level by the surveillance focal person, while the non-priority diseases were reported monthly to the county data managers. However, the immediately reportable diseases according to IHR were reported in a shortest time possible after identification. During 2017, 87% (4364/5016) of the priority diseases cases notified were linelisted and 86% (4314/5016) were notified from the facility to district level within 24 hours (Figure 2).

**Data management:** the data collected in the communities and from the entry points were submitted in hard copies to the HCFs and the immediate reportable diseases data were stored in both hard copies and DSOs computers to be submitted every week to the county level. The other conditions were sent to the county level in hard copies every month. At the county level, all the reports were compiled, stored in the computers and submitted to the national level. While the immediate reportable diseases were submitted in excel sheets, the other diseases were submitted through health management information system (HMIS). In 2017, the country piloted the use of mobile phones application [Open Data Kit (ODK) ©] to store and send data from the HCFs to the national level by the DSOs. Each level was retains one copy of the reports submitted to the next level for future inspections. An electronic platform for acute flaccid paralysis (AFP) surveillance called Auto-Visual AFP Detection and Reporting (AVADAR) was piloted in Montserrado County in 2017 and electronic IDSR piloted in Margibi and Grand Cape Mount Counties. One of the OICs highlighted the need of harmonization of HMIS and IDSR from the HCF perspective as a way to improve IDSR data management at HCF level.

**Data analysis:** at the county level, the data was presented in graphs and tables by the county surveillance officer (CSO) and WHO epidemiologists. All the 15 county surveillance officers analyzed data at least once a month and presented the findings to the county health team’s supervisors and partners during the monthly county surveillance meetings. The NPHIL produced 52 weekly bulletins and one semester bulletin of priority diseases and events in 2017. Some OICs mentioned that it was difficult to make regular graphs for the priority diseases in the HCFs.

**Outbreak preparedness:** after the EVD outbreak, the incidence management system was successfully reactivated during an outbreak of meningococcal disease in 2017 at both national and county level. The infection prevention and control (IPC) protocols were available at 82% of the HCFs supervised in 2017. At county level, there were county rapid response teams (CRRT) and at district level there were districts rapid response teams (DRRT), both trained during and after EVD outbreak. The lack of isolation and triage at the HCFs were some of the major challenges identified by OICs during the supervisions. In addition, they reported constant stock out of IPC materials and inadequate referral system.

**Outbreak detection:** the linelist of alerts and rumors from the community were available in 46% of the HCFs assessed in 2017. From January to December, 2017, the country detected suspected outbreaks of 18 different conditions (Table 2 and Table 3). From those conditions, cases of shigellosis, human rabies, cholera, Lassa fever, measles, rubella and meningococcal disease were confirmed by national and international labs (Table 2). Some conditions were confirmed clinically, with no lab tests performed (Table 3). All the suspected measles cases with results negative were tested for rubella and 315 positives cases were detected from all the regions. We identified some sporadic comprehensive outbreaks investigation reports at the national level received from the counties, especially for meningococcal disease, measles, lassa fever, skin diseases and cholera.

**Table 3:** Outbreaks investigated with no cases confirmed by laboratory, Liberia, January to December, 2017

	**South Eastern A**			**South Eastern B**			**North Western**			**South Central**			**North Central**			**Liberia**		
	**Cases**	**Deaths**	**CFR (%)**	**Cases**	**Deaths**	**CFR (%)**	**Cases**	**Deaths**	**CFR (%)**	**Cases**	**Deaths**	**CFR (%)**	**Cases**	**Deaths**	**CFR (%)**	**Cases**	**Deaths**	**CFR (%)**
**Acute Flaccid Paralysis ^a^**	97	1	1.0	5	0	0.0	11	0	0.0	34	0	0.0	104	0	0.0	251	1	0.4
**Chicken pox^b^**	2	0	0.0	1	0	0.0	0	0	NA	14	0	0.0	0	0	NA	17	0	0.0
**Monkey pox^c^**	1	0	0.0	1	0	0.0	2	0	0.0	0	0	NA	0	0	NA	4	0	0.0
**Neonatal Tetanus^b^**	1	1	100.0	3	0	0.0	3	0	0.0	13	4	30.8	2	0	0.0	22	5	22.7
**Pertussis^b^**	110	0	0.0	10	0	0.0	0	0	NA	72	0	0.0	0	0	NA	192	0	0.0
**Scabies^b^**	7	0	0.0	42	0	0.0	3	0	0.0	0	0	NA	0	0	NA	52	0	0.0
**Skin rashes ^b^, ^d^**	38	0	0.0	0	0	NA	0	0	NA	0	0	NA	0	0	NA	38	0	0.0
**Unexplained cluster of Health event^d^**	3	0	0.0	10	0	0.0	0	0	NA	16	3	18.8	0	0	NA	29	3	10.3
**VHF(including EVD)^e^**	53	32	60.4	56	47	83.9	17	15	88.2	70	64	91.4	99	81	81.8	295	239	81.0
**Yellow fever^f^**	3	0	0.0	39	0	0.0	45	0	0.0	11	0	0.0	5	1	20.0	103	1	1.0
**Unexplained Cluster of Death^d^**		6			3			0			2			0			11	
**Maternal death**		25			22			14			90			74			225	
**Neonatal death**		41			133			20			242			144			580	

a The Acute flaccid paralysis cases were tested for poliomyelitis and all the results came negative;

b Outbreak was confirmed clinically with no lab test performed;

c Outbreak was not confirmed with no lab test performed;

d There was no conclusion about the nature of the event

e The outbreak was not confirmed after all the lab results test negative for viral hemorrhagic fever (VHF) including Ebola virus disease;

f The rapid test performed in Liberia was positive for yellow fever but the confirmatory test performed in Senegal came negative and the outbreak was not confirmed

**Outbreak response:** according to the records reviewed, all the outbreaks detected in 2017 were on average investigated within 48 hours, accompanying initial investigation reports. The main outbreaks (measles, lassa fever and meningococcal disease) had regular situational reports from the counties and national level. The response of the outbreaks varied from local responses for the small outbreaks such as scabies, pertussis, shigellosis, to complete reactivation of incident management system (IMS) at the county and national level for outbreaks of meningococcal disease, lassa fever and measles. The meningococcal disease outbreak was responded with chemoprophylaxis (ciprofloxacin and ceftriaxone) among other measures, lassa fever was responded to with appropriate case management (treatment with ribavirin), contact tracing and isolation of the cases and measles campaigns and administration of vitamin A were performed to control the measles outbreaks. The other outbreaks were also controlled according to the situation with emphasis to health education, sanitation and hygiene, besides case management and immunization for vaccine preventable diseases like yellow fever.

**Laboratory function:** some laboratories with capacity to confirm EVD (RT-PCR, GeneXpert), Lassa fever, Yellow fever, acute watery diarrhea, acute bloody diarrhea and meningitis were installed at the national level and strategic counties from 2015 to 2017. The stool specimens were tested for poliomyelitis in Ivory Coast, while the yellow fever cases were confirmed in Senegal and lassa fever in Sierra Leone. The other conditions were confirmed in other countries based in previous negotiation. For instance, the rabies cases was confirmed in France and meningococcal disease had specimens tested in Atlanta, United States, France and South Africa. A total of 60 couriers from Riders-for-Health were transporting specimens from 302 HCFs across the country to the public health laboratories from January to June 2017. During the same period, a total of 1631 specimens were tested for 8 epidemic-prone diseases and conditions. The lab results were sent regularly by email of the county health teams (CHT) created specifically for lab results to be assessed by key CHT staff and partners. The frequent stock out of lab supplies including specimen collection materials, lack of fridge to store the specimen in one HCF and inadequate lab space in other HCF were the main challenge reported by OICs during the supervisions.

**Data management and feedback:** at the national level, the data were stored in excel sheet for IDSR data and DHIS2 for the monthly reportable diseases. All the bulletins produced by NPHIL were published at the MOH and NPHIL websites. A feedback power point presentation was conducted weekly by NPHIL to partners and stakeholders. From the national to county level, power point feedback on IDSR situation in Country and lab results were circulated through email weekly. There was no evidence of any feedback provided about the monthly reportable diseases at all levels. At the county level, the weekly IDSR supervisions were used to provide feedback from the county to district and facility level. Although VHF-Epi-info database was being used for EVD outbreak in a parallel surveillance system, later integrated into the HMIS (DHIS2) there is no documentation of other surveillance systems used before the implementation of IDSR. The OICs were not satisfied with feedback received, and because the issues from previous supervisions were rarely or never addressed.

### Support functions

**Trainings:** in 2017, all the county, district and zonal surveillance officers were trained in IDSR and field epidemiology training program (FETP). In addition, different refresher IDSR trainings were conducted targeting all the health workers and stakeholders across the country. Nevertheless, during the 2017 assessment, only 46% of the IDSR focal persons from the facilities (OICs) were trained in IDSR in the past one year and 76% of the HCFs had at least one staff trained in swab collection. However, 84% of the facilities had community volunteers in the catchment areas trained in community based surveillance in the past one year. The IDSR training at all levels was supported by WHO, while other partners were involved also in IDSR training at the community level. The inadequate training of the HCF staff including the OICs was the most important challenge identified by a considerable number of OICs supervised. They mentioned the need of training of other staff including vaccinators, since the OICs is usually overwhelmed with other activities. Other solutions suggested were regular supportive supervisions, in-site mentoring and retain the trained staff in the HCF.

**Supervisions:** the IDSR supervisions were conducted regularly in Liberia where the HCFs should be supervised at least once per month. From September to November 2017, 50% of the HCFs were supervised, with the highest percentage (89%) in south western region and lowest percentage in the south eastern B (26%). On the other hand, 93% of all the HCFs received supervision feedback report from district/county in the past four weeks and 82% of the facilities conducted regularly supervisions to the community volunteers.

**Resources:** in 2016, WHO provided motorbikes, computers and printers to all the DSOs, CSOs and ZSOs and office equipment to all the district health teams (DHTs) across the country. Besides fuel, WHO also provided lubricants, spark plugs, spare parts for motorbikes and scratch cards for communication provided to all the districts every quarter. However, although 94% of the HCFs were supervised from September to November 2017, 42% had no adequate and functioning transport, including fuel, for surveillance activities. The inadequate resources to perform the surveillance activities at the facility level was one of the most important challenges raised by the OICs. They requested motorbikes (including maintenance), fuel support, phone and scratch cards for communication, and financial incentives to encourage the overwhelmed staff to perform surveillance activities. One OICs requested one more clinician to support the OIC since the clinic was operating with only two staff.

**Standards and guidelines:** although from 2015 to 2017, two versions of IDSR guidelines were produced in Liberia and distributed to all the HCFs, during our 2017 assessment 34% of the facilities had no IDSR guidelines available during our assessment. The standard operation procedures (SOPs) for dead body management was available in 74% of the facilities while the SOPs for sample collection, packaging, and storage for priority diseases were present in 76% of the HCFs. One of the OICs interviewed requested to be supplied with additional IDSR guidelines.

**Coordination:** from 2015 to 2016, all the IDSR activities in Liberia were coordinated by MOH and taken over by NPHIL after its creation in 2017. NPHIL is directly supported by MOH and partners including WHO and Center for Disease Control and Prevention. The DSOs who were identified, trained and assigned in 2016 were responsible for coordinating the IDSR activities at the district level, while the CSOs, who were previously part of the MOH system coordinated the IDSR at the County level. During the organizational stage of the NPHIL, the DSOs incentives were provided directly by WHO. However, when the payment was handed over to NPHIL, there was a temporary interruption of payment which led to dissatisfaction and temporary refusal of the DSOs and ZSOs to submit reports to the national level, although the surveillance activities never were interrupted.

## Discussion

Our study shows that IDSR has been actively implemented in Liberia since 2015. Feedback to the counties and HCFs, including laboratory results using email, regular supervision to all the HCFs, the introduction of mobile phone applications for data collection and management and the electronic platform for AFP surveillance were some of the innovations observed during this period. The electronic surveillance is useful in reducing the time from detection to reporting of public health events, allowing a fast investigation and response [14]. Although the electronic surveillance was piloted in Liberia, a study conducted in Tanzania in 2012 [15] and Kenya in 2016 [16] demonstrated that the use of mobile phones for surveillance can dramatically improve the timeliness and completeness of the reports. The reestablishment of IDSR in Liberia utilized the existing health structure through integration of existing surveillance systems. This included establishment of a coordination mechanism to link various surveillance systems to create an integrated system, harmonization of data collection tools, procurement of standardized data storage; and storing data in a uniform database where could easily be accessed by users and policy makers. This integrated system is particularly important to address all the public health events notifiable under IHR (2005) [17]. However, Liberia was running a parallel system (HMIS) to report monthly other conditions together with the conditions reportable under IDSR, leading to data discrepancies. The same challenge was observed during an assessment conducted in Ethiopia [18] and was even worse in Tanzania in 1998, when five parallel surveillance systems lead to poor performance in all the IDSR functions [19].

The implementation of IDSR in Liberia guided the decision-making for public health action and contributed to the overall health sector goal of reducing morbidity and mortality due to preventable causes, exemplified by all the outbreaks suspected, confirmed and controlled quickly with law case fatality rate in 2017. However, although malaria was the most important cause of admission and deaths in Liberia, especially among children [12, 20], it was not part of IDSR. In other countries like Ghana [21] and Ethiopia [18], the use of malaria data obtained through IDSR lead to important decisions to reduce the morbidity and mortality of the illness. The resilience of surveillance system in Liberia was also contributed by the integration of the private HCFs into IDSR, considering that 38% (296/773) of the HCFs in Liberia were private, with 96% (285/296) of them reporting regularly to the surveillance system. Unlike Liberia, the integration of private facilities into surveillance system in other countries was a challenge, compromising the completeness of reporting [13]. Despite the temporary interruption in reporting by the DSOs and ZSOs verified in 2017 in Liberia, the completeness of reporting remained high in 97%, since the private facilities continued reporting and ZSOs from Montserrado were supported by other partners. However, it affected negatively the number of supervisions conducted to the HCFs, being 50% (384/773) from September to November, 2017, far from the 80% recommended in IDSR guidelines. The consistent high completeness and timeliness of reporting was also contributed by the availability of resources like computer, printers and means of transport provided by WHO at district and county level. However, the same resources still not available at facility level in Liberia. The lack of resources, including reporting forms verified in Nairobi [22] and Nigeria [23] compromised the submission of weekly reports and other IDSR functions in general.

Although all the HCFs in Liberia had IDSR focal person, the follow up trainings were poor, representing only 46% (178/384) of IDSR focal point trained in the past year due to attrition of the previously trained staff. This may lead to poor quality of reporting, difficulties to use the standard case definition (relying more in clinical judgment), poor data analysis skills and inadequate supervisions and feedback to the community level as demonstrated in Tanzania in 2002 [24]. Liberia also had not integrated yet into IDSR other components of IHR (2005) like animal, food poisoning, chemical poisoning and disaster in the same level as other countries [17]. Instead, those events were classified as cluster of events and deaths. The qualitative questions from IDSR supervision revealed that the health workers were not satisfied with their level of training in IDSR, besides the workload, poor feedback with the issues raised in the supervision not addressed before the following supervision and inadequate means of transport and communication. The same findings were observed in qualitative study conducted in Zambia in 2016 [25]. Our study had several potential limitations. There was no information available about the implementation of IDSR in Liberia before the EVD outbreak. In addition, after the re-introduction of IDSR in 2015, different supervision tools were used, making difficult to compare the progress of indicators over the years. The assessment was also done by people from the county (DSOs) and information biases are expected. The interviews with the OICs were not recorded and transcribed, making impossible to reproduce verbatim quotes in our analysis. Despite these limitations, our findings can reliably be used for decision making and documentation.

## Conclusion

Liberia is facing the same challenges as other countries during the early stages of implementation of IDSR. However, the innovations were implemented in a short time. The surveillance system was resilient, despite the challenges. The resources are available at national, county and district level, but inadequate at the facility level. The supportive supervisions were conducted regularly but the health workers at facility level were not satisfied with the feedback provided to them. We recommend the government and partners to give attention to the HCFs, especially providing adequate resources and regular feedbacks.

### What is known about this topic

- Integrated diseases surveillance and response is actually being implemented in several countries in Africa, including Liberia;
- Liberia adapted the integrated diseases surveillance and response (IDSR) in 2004 as a platform for implementation of International Health Regulation (IHR (2005));
- Liberia and other west african countries recognized late the Ebola virus diseases outbreak in 2013/2014 after several deaths being reported.

### What this study adds

- Status of integrated disease surveillance and response implementation in Liberia to avoid deadly diseases outbreaks to be recognized late and avoid high cases fatality rate;
- Best practices on implementation of integrated diseases surveillance and response in Liberia after the Ebola virus diseases outbreak;
- Challenges faced by the country to implement the integrated diseases surveillance and response after the Ebola virus disease outbreak.

## Competing interests

The authors declare no competing interest.
